# Evaluation of parameters for fetal behavioural state classification

**DOI:** 10.1038/s41598-022-07476-x

**Published:** 2022-03-01

**Authors:** Lorenzo Semeia, Katrin Sippel, Julia Moser, Hubert Preissl

**Affiliations:** 1 IDM/fMEG Center of the Helmholtz Center Munich at the University of Tübingen, University of Tübingen, German Center for Diabetes Research (DZD), Otfried-Müller-Str. 47, 72076 Tübingen, Germany; 2 Graduate Training Centre of Neuroscience, International Max Planck Research School, University of Tübingen, Tübingen, Germany; 3 Department of Internal Medicine IV, University Hospital of Tübingen, Tübingen, Germany; 4 Department of Pharmacy and Biochemistry, Interfaculty Centre for Pharmacogenomics and Pharma Research, University of Tübingen, Tübingen, Germany

**Keywords:** Computational biology and bioinformatics, Physiology

## Abstract

Fetal behavioural states (fBS) describe periods of fetal wakefulness and sleep and are commonly defined by features such as body and eye movements and heart rate. Automatic state detection through algorithms relies on different parameters and thresholds derived from both the heart rate variability (HRV) and the actogram, which are highly dependent on the specific datasets and are prone to artefacts. Furthermore, the development of the fetal states is dynamic over the gestational period and the evaluation usually only separated into early and late gestation (before and after 32 weeks). In the current work, fBS detection was consistent between the classification algorithm and visual inspection in 87 fetal magnetocardiographic data segments between 27 and 39 weeks of gestational age. To identify how automated fBS detection could be improved, we first identified commonly used parameters for fBS classification in both the HRV and the actogram, and investigated their distribution across the different fBS. Then, we calculated a receiver operating characteristics (ROC) curve to determine the performance of each parameter in the fBS classification. Finally, we investigated the development of parameters over gestation through linear regression. As a result, the parameters derived from the HRV have a higher classification accuracy compared to those derived from the body movement as defined by the actogram. However, the overlapping distributions of several parameters across states limit a clear separation of states based on these parameters. The changes over gestation of the HRV parameters reflect the maturation of the fetal autonomic nervous system. Given the higher classification accuracy of the HRV in comparison to the actogram, we suggest to focus further research on the HRV. Furthermore, we propose to develop probabilistic fBS classification approaches to improve classification in less prototypical datasets.

## Introduction

In 1974 Prechtl described for the first time ‘behavioural states’ in infants^1^, which is a concept that was later applied to fetuses^2^. Starting from 32 weeks of gestational age (GA), four fBS were identified, namely quiet sleep (1F), active sleep (2F), quiet awake (3F) and active awake (4F). The definition criteria of these states rely on body/eye movement and heart rate patterns that are stable for at least 3 min (see Table 1). Fetal states can be detected even before 32 weeks of GA but, in this case, it is only possible to define periods of rest (quiet states, QS) and periods of activity (active states, AS;^3^. The distribution of these fBS has been found to be stable over daytime^4^ but depends on maternal position^5^.

**Table 1:** Fetal behavioural states original criteria defined by Nijhuis et al. in 1982^2^.

	State 1F	State 2F	State 3F	State 4F
Body movements	Incidental	Periodic	Absent	Continuous
Eye movements	Absent	Present	Present	Present
Heart rate patterns	Stable heart rate, with small oscillation bandwidth. Accelerations strictly related to movement	Wider oscillation bandwidth compared to 1F. Frequent accelerations related to body movements	Stable heart rate. No acceleration and higher oscillation bandwidth compared to 1F	Unstable heart rate. Long lasting accelerations, often with tachycardia

Fetal states were investigated in relation to fetal health^6^, fetal maturation^7^ and the development of the autonomic nervous system (ANS)^8,9^, as well as in fetal cognitive processing^10^. With the advancement of technology, it was possible to correctly classify states even without the usage of all defined indicators.

For example, Maeda described^11^ how it was possible to define fBS without considering eye movements, adopting only the fetal heart rate and the gross body movement as available in the actocardiogram.

This also enabled the investigation of fBS using fetal magnetocardiography (fMCG). This device allows to non-invasively record the magnetic signal of the fetal heart directly through the maternal abdomen. Compared to classical CTG and ultrasound, fMCG provides a higher temporal resolution and it is less susceptible to maternal interferences^12^. These recordings provide more accurate fetal heart dynamic parameters, like heart rate variability (HRV) measures.

The gold standard for fBS classification using fMCG is a visual inspection and classification of the fetal HRV and actogram by experts. To facilitate this classification, the original Nijhuis criteria (^2^, see Table 1) were adapted by Brändle et al. in 2015 (^9^, see Table 2). Due to the low occurrence of state 3F in this adaption only states 1F, 2F, and 4F were included. The fetal actogram describes the movement of the fetal body by calculating the spatial location of the highest heart activity amplitude (heart vector) for every single heartbeat, and follows the changes of this location within the sensor array over time.

**Table 2:** Fetal behavioural states definition criteria by Brändle et al. from 2015^9^.

	1F	2F	4F
Baseline	< 160 bpm	< 160 bpm	> 160 bpm possible
Oscillation bandwidth	≤ ± 7.5 bpm	± 7.5 – ± 15 bpm	> ± 15 bpm
Accelerations	No	> 15 bpm/ > 15 s	> 30 bpm/ > 30 s
Movement	No	Yes	Yes

Recently, some algorithms have been developed to automatically classify fBS, both in CTG^13^ and in fMCG^14^. Therefore, the original criteria defined by Nijhuis et al.^2^ had to be translated into values that could be used as classification thresholds by those algorithms.

To achieve the quality and accuracy of a visually-inspected evaluation performed by experts, automatic state detection algorithms rely on the combination of several heart and movement parameters and their more or less strictly defined thresholds. These thresholds are highly dependent on the specific datasets and, although this is a practical solution for the study of prototypical fetal physiology during the states, they are only applicable to those datasets in which no artefacts are occurring. Moreover, given the dynamic development of fBS over gestation, for classification purposes researchers usually separate the early and the late gestation (before and after 32 weeks of GA). Altogether, these factors make it difficult to obtain a satisfactory classification of fBS. In the current work we aim to perform an exploratory analysis of commonly used fBS classification parameters, like variability in the heart-rate or in the actogram, to determine the reliability of these parameters. Secondly, the development of these parameters over gestation is investigated.

The first part (Step1, “Parameter distribution and thresholds”) of the current work will investigate the accuracy of the fBS classification parameters. Therefore, we analysed the distribution of the main parameters commonly used in fBS algorithms.

Second (Step2, “Best classifier”), we want to verify which of the heart-rate (HR) or movement parameters are the best parameters for state classification performance both in early as in late gestation.

Finally (Step3, “Changes over time”), we investigate changes and development of these parameters over the last trimester of gestation, in order to highlight possible age-dependent trends.

## Methods

### Fetal magnetocardiography

The fetal magnetocardiographic data used in the current work are recorded using a fetal Magnetoencephalography (fMEG) device. fMEG is a non-invasive device for the recording of brain and heart activity of fetuses in the last trimester of gestation, and of newborns in the first weeks of life^15^. The datasets included in the current study were recorded using a SARA (SQUID Array for Reproductive Assessment, VSM MedTech Ltd., Port Coquitlam, BC, Canada) system available at the fMEG Center at the University of Tuebingen, Germany. All the recordings were sampled with a frequency of 610,3516 Hz. This device comprehends 156 magnetic sensors and 29 additional reference sensors. These sensors are placed in a concave array, designed to match the maternal abdomen. In order to limit the influence of external magnetic fields, the device is placed in a magnetically shielded room (Vakuumschmelze, Hanau, Germany).

### Datasets

The 169 original fMEG datasets (117 for fetuses younger than 32 weeks of GA) used in the current analysis come from previously published studies^16,17^. All recordings were performed in the morning. In addition, previous data showed that the fBS distribution is stable over daytime^4^. These datasets were previously screened for good quality of the raw data in terms of absence of large amplitude artefacts. These studies were approved by the Ethical Committee of the Medical Faculty at the University of Tuebingen (No. 476/2008MPG1 and 339/2010BO1). All the participants to these studies gave their informed consent in accordance to the Declaration of Helsinki and agreed for the usage of the data in further studies. The datasets include recordings of spontaneous activity as well as recordings during an auditory paradigm. The datasets length varies between 6 and 15 min.

### Preprocessing

The data preprocessing was performed using Matlab R2016b (The MathWorks, Natick, MA, USA). R-peak detection of maternal and fetal heart has been performed using FLORA^18^. Maternal heart interference was removed by FAUNA^19^. HRV parameters in the time domain (mean heart-rate (HR), the standard deviation of normal to normal R-R intervals (SDNN) and the root mean square of successive differences between normal heartbeats (RMSSD) were calculated for the fMCG signals^20^. Finally, the fetal actogram and cardiogram were calculated as described by Govindan et al.^21^. The actogram describes the spatial location of the highest fetal cardiac signal in relation to the magnetic sensors for each time point, and is therefore a measure for fetal activity and movement.

### Fetal states analysis

The fetal behavioural state underlying the datasets was automatically detected using an algorithm adapted from the work of Vairavan et al.^14^ and implemented in Matlab R2019b.

We tested the algorithm on three groups based of the fetal gestational age (GA), an early, a middle, and a late group. The early group includes datasets from fetuses between 27 and 32 (27 0/1–32 6/7) weeks of GA, the middle group fetuses between 33 and 36 (33 0/1–36 6/7) weeks of GA, and the late group includes fetuses between 37 and 39 (37 0/1–39 6/7) weeks GA. According to the work of Vairavan et al.^14^, the percentage of accelerations in the HR, the standard deviation of the HR (σ(HR)) and the percentage of points above 160 bmp were computed. The thresholds for these values used in our algorithm are also from Vairavan and collaborators^14^. To differentiate between active and passive states in fetuses from 27 to 29 (27 0/1–29 6/7) weeks of GA we applied the threshold of 5.54% of accelerations for dividing between passive (≤ 5.54%) and active (> 5.54%) states. This threshold is based on Vairavan et al.^14^, where it is used for dividing between active and passive states between 30 and 36 (30 0/1–36 6/7) weeks of GA.

Out of the included 169 datasets, a state was classified for approximately 80% of the total data length. In the remaining 20% of data, the variability of parameters used for classification (e.g. the percentage of acceleration in the HR) was exceeding the tolerance of the detection algorithm, thus preventing a fBS classification. This variability is likely due to transitions between states or to signal noise.

To maintain a reasonable amount of data for each group, we considered the middle and late group as a whole late group for all the further analyses. From the algorithm outputs, 120 data segments (from 85 different recordings performed in 52 subjects) were selected based on a clear detection of the behavioural state: 31 for active and 27 for passive in the early group, 31 for 1F and 31 for 2F in the late group. The goal of the selection was to have an equivalent amount of datasets in each group however, no more than 27 passive state windows were available. Yet, a lower occurrence of passive states is expected^22^. 3F and 4F were not included because we were not able to detect enough data segments. The mean duration of these 120 data segments was 266 ± 44 s.

The data segments were then visually evaluated by two experts to gain security about the result of the automatically classified states. There was agreement between the two raters in 96 windows (80% agreement). 87 out of these 96 windows were also in agreement with the automatic states classification algorithm. Following this procedure, our further analysis only contains those 87 datasets in which there was accordance between all 3 ratings (see Table 3).

**Table 3:** Characteristics of the 87 data segments that were chosen for further evaluation [mean ± standard deviation].

	Passive	Active	1F	2F
Windows length, in seconds	288.63 ± 30.83	252.81 ± 43.98	283.60 ± 34.07	250.00 ± 52.98
Number of windows	19	31	15	22
GA, in weeks	29.21 ± 1.47	29.52 ± 1.43	35.13 ± 1.41	34.95 ± 1.65

### Parameters definition

The following parameters, which are based on the states definition criteria reported in Table 1 and Table 2, are included in the further analysis. For a summary of the parameters see Table 4.

**Table 4:** Summary of the parameters used in the current work.

HRV	Actogram
STD-baseline	RMSSD-actogram
% points outside ± 5 bpm	STD-actogram
% points outside ± 7.5 bpm	
RMSSD-HR	
STD-HR	
Correlation between cardiogram-actogram	

Classical HRV parameters (mean HR, SDNN-RR and RMSSD-RR) are calculated on the RR intervals in order to verify whether their values are comparable to previous work which investigated fBS^9^. Therefore, their classification accuracy and development over time are not further investigated.

In the HRV we defined A) Baseline standard deviation (*STD-baseline*). The baseline is defined as smoothing the HR in a two minutes moving window with 1 s shift. We considered the baseline standard deviation as an index of variability in the heart rate. B) Percentage of points outside the ± 5 bpm oscillation bandwidth (*% points outside* ± *5 bpm*), based on Nijhuis et al.^2^. The oscillation bandwidth is defined as the area between the baseline and the baseline ± 5 bpm. C) Percentage of points outside the ± 7.5 bpm oscillation bandwidth (*% points outside* ± *7.5 bpm*) based on Brändle et al.^9^. The oscillation bandwidth is defined as the area between the baseline and the baseline ± 7.5 bpm. D) Root mean square of successive differences in the HR (*RMSSD-HR*). This parameter has been chosen as an index of short-term variability in the heart rate. In contrast to the classical HRV parameter RMSSD, which is calculated on the time differences between successive R peaks, this parameter is directly calculated on HR values. E) Heart rate standard deviation (*STD-HR*). This is an index of variability in the heart rate.

In the actogram we defined F) Root mean square of successive differences in the actogram (*RMSSD-actogram*). This parameter has been selected as an index of short term variability in the actogram. G) Actogram standard deviation (*STD-actogram*). This is an index of variability in the actogram.

Finally, since in the original definition by Nijhuis et al.^2^ fBS are also defined based on the relation between heart-rate and movement accelerations, we calculated the correlation between cardiogram and actogram to explore whether this parameter could be used for states classification.

### Statistics

Statistics were performed with Matlab R2019b for Windows. Before proceeding, outliers for each of the parameters, defined as a value exceeding the mean ± 3 standard deviations, were removed. Then, the Kolmogorov–Smirnov test was used to test the normality assumption required by the following statistical analysis.

### Step 1. Parameter distribution and thresholds

We tested the hypothesis that there is a difference between HRV, cardio- or actogram parameters between active and passive (H1: μActive—μPassive ≠ 0), or between 1 and 2F (H1: μ2F—μ1F ≠ 0), state.

A t-test was performed for the analysis of the different parameters between active and passive, and between 1 and 2F, state After Bonferroni correction for N = 10 different parameters, the significance level was set to p < 0.005.

### Step 2. Best classifier

Receiver operating characteristics (ROC) curves were defined to verify the performance of each parameter in classifying a state.

### Step 3. Changes over time

Finally, to detect the development of parameters during gestation, linear models were built for each parameter. In the models, we combined passive (early passive + 1F) and active (early active + 2F) states between 27 and 39 weeks of GA. The model was defined as:

$$y = \beta 0 + \beta 1{\text{X }} + \varepsilon$$

where *y* is one of the parameters included in the current analysis, β0 is the intercept, β1 the regression coefficient, X the gestational age, and ε the error in the estimate. Model values of p < 0.05 were considered statistically significant and effect size, defined by r^2^, was calculated.

## Results

### Step 1. Parameters distribution and thresholds

#### HRV parameters

**Figure 1:**
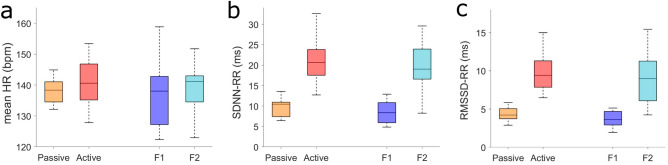
Box and whiskers plots of the HRV parameters in the four defined states. Depicted are the median (central mark), and the 25th and 75th percentile (bottom and top edges of the box, respectively). (**a**) Heart rate in beats per minute (bpm). (**b**) Standard deviation of normal to normal R-R intervals, in milliseconds (ms). (**c**) Root mean square of successive differences between normal heartbeats, in milliseconds (ms).

The t-test between states revealed no difference in the mean HR both between passive and active state in the early gestation (t(48) = 1.46, p = 0.15), and between 1 and 2F in the late gestation (t(35) = 0.38, p = 0.70) (Fig. 1a). SDNN-RR was higher in active compared to passive state (t(45) = 9.20, p < 0.001), and higher in 2F compared to 1F state (t(35) = 7.36, p < 0.001) (Fig. 1b). Finally, RMSSD-RR was higher in active compared to passive state (t(48) = 10.43, p < 0.001), and higher in 2F compared to 1F (t(35) = 6.40, p < 0.001) (Fig. 1c).

Although two out of three HRV parameters showed a significant difference both in early and in late gestation, only the RMSSD-RR values in the early gestation could be separated by a single threshold. The distributions of all other parameters overlap.

#### Cardio- and actogram parameters

In the early gestation, the t-tests revealed that all parameters were higher in active compared to passive states: ‘STD-baseline’ (t(45) = 5.59, p < 0.001), ‘% points outside ± 5 bpm’ (t(46) = 13.47, p < 0.001), ‘% points outside ± 7.5 bpm’ (t(46) = 14.16, p < 0.001), ‘RMSSD-HR’ (t(42) = 13.54, p < 0.001), ‘STD-HR’ (t(44) = 13.80, p < 0.001), ‘RMSSD-actogram’ (t(43) = 3.46, p = 0.001), and ‘STD-actogram’ (t(42) = 4.22, p < 0.001).

Also in late gestation, the t-tests revealed that all parameters were higher in 2F compared to 1F: ‘STD-baseline’ (t(34) = 5.28, p < 0.001), ‘% points outside ± 5 bpm’ (t(32) = 16.73, p < 0.001), ‘% points outside ± 7.5 bpm’ (t(35) = 15.91, p < 0.001), ‘RMSSD-HR’ (t(32) = 6.97, p < 0.001), ‘STD-HR’ (t(32) = 11.65, p < 0.001), ‘RMSSD-actogram’ (t(32) = 3.67, p < 0.001), and ‘STD-actogram’ (t(34) = 4.07, p < 0.001).

**Figure 2:**
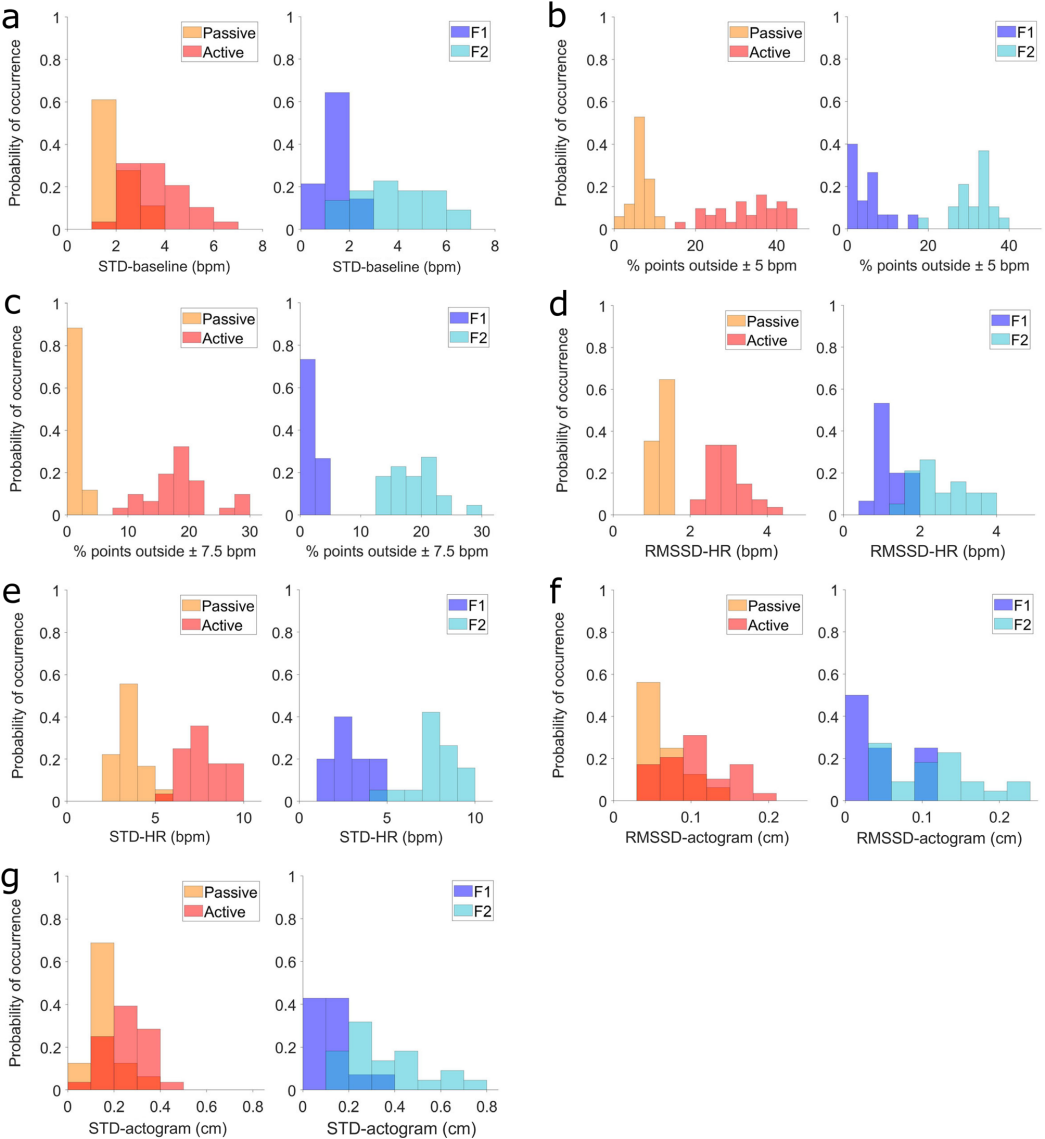
Parameters distributions divided by gestational age. (**a**) STD-baseline (bpm). (**b**) % points outside ± 5 bpm. (**c**) % points outside ± 7.5 bpm (%). (**d**) RMSSD-HR (bpm). **e**) STD-HR (bpm). (**f**) RMSSD-actogram, in centimeters (cm). (**g**) STD-actogram, in centimeters (cm).

Although all of the seven cardio- and actogram parameters show significant differences in their distribution in early and late gestation, only three parameters in early and two in late gestation could be separated by a single threshold, while the distributions of all the other parameters overlap (see Fig. 2).

For details about the mean parameters values, see supplementary table 1 and supplementary table 2.

#### Correlation cardiogram-actogram

**Figure 3:**
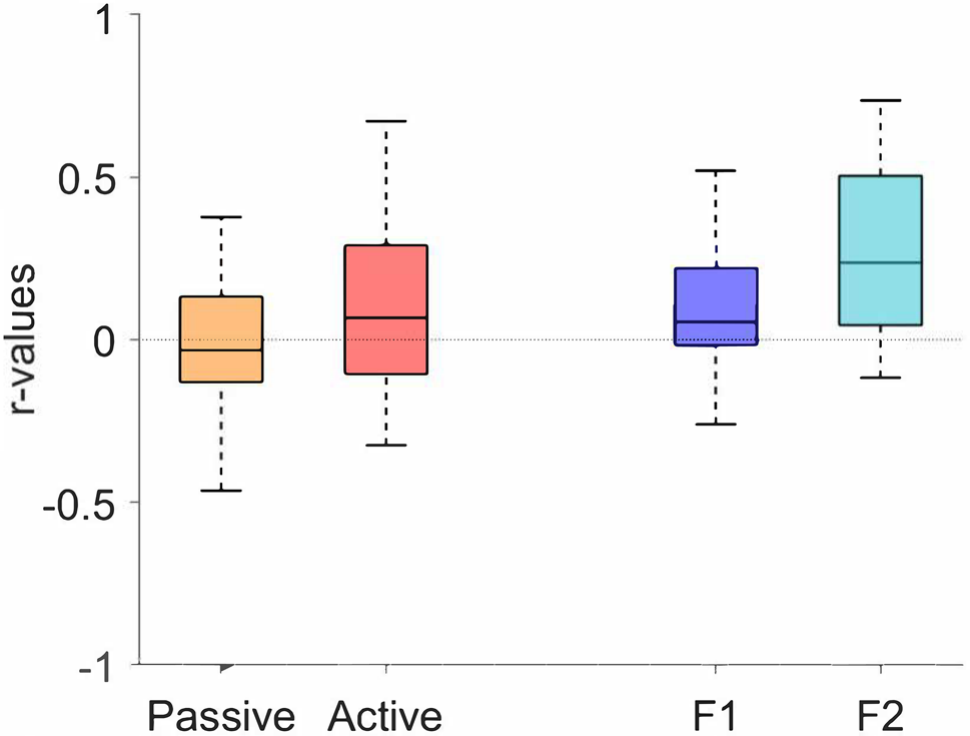
Correlations between cardiogram-actogram. Depicted are the median (central mark), and the 25th and 75th percentile (bottom and top edges of the box, respectively).

Correlating the cardiogram and the actogram, the t-tests revealed no difference in the mean r-value between passive (M = -0.01, SD = 0.22) and active (M = 0.08, SD = 0.26) state in the early gestation (t(48) = 1.32, p = 0.19). There was a significant difference between 1F (M = 0.09, SD = 0.21) and 2F (M = 0.28, SD = 0.27) in the late gestation (t(35) = 2.33, p = 0.03) (see Fig. 3).

### Step 2. Best classifier

#### ROC analysis

**Figure 4:**
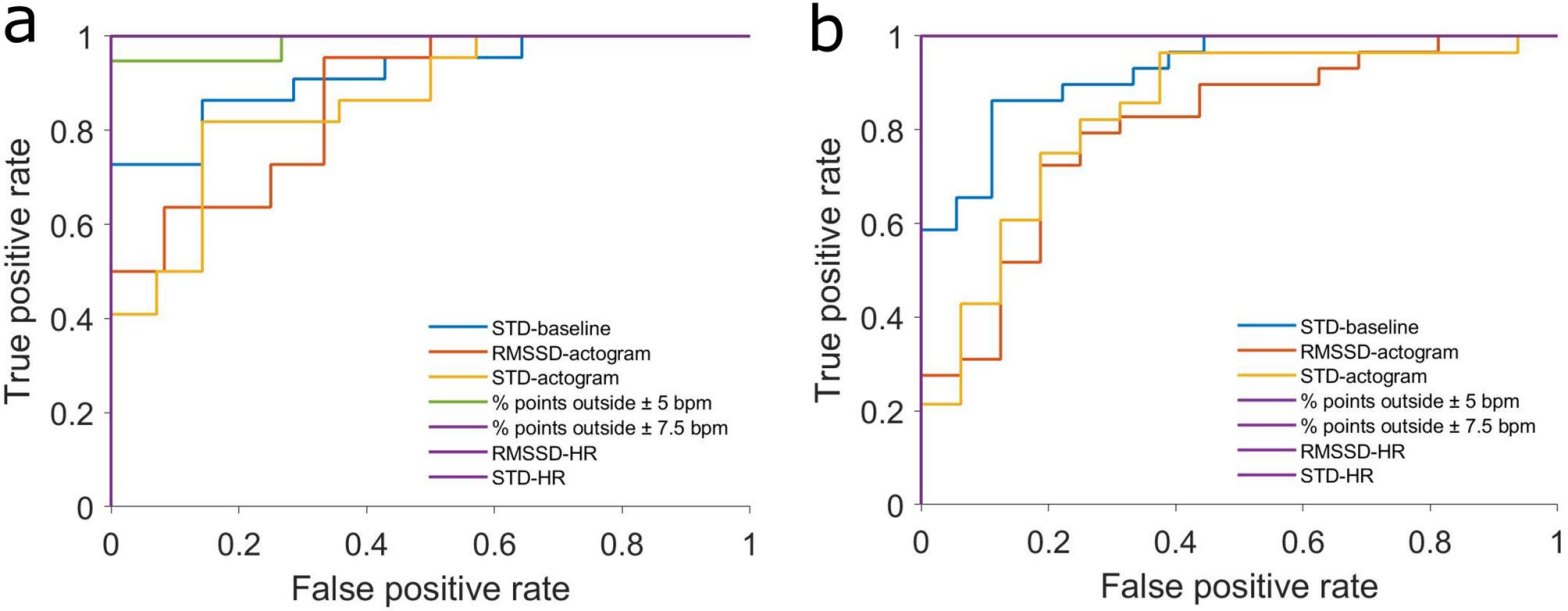
Receiver operating characteristics (ROC) curve for (**a**) early gestation and for (**b**) late gestation.

In the early gestation, ‘% points outside ± 5 bpm’, ‘% points outside ± 7.5 bpm’, ‘STD-HR’ and ‘RMSSD-HR’ are perfect classifier (AUC = 1), ‘STD-baseline’ has a high performance (AUC = 0.93), ‘RMSSD-actogram’ (AUC = 0.80) and ‘STD-actogram’ (AUC = 0.83) perform less compared to the other classifier (see Fig. 4a).

In the late gestation the results are very similar. ‘% points outside ± 5 bpm’, ‘% points outside ± 7.5 bpm’, ‘STD-HR’ are perfect classifier (AUC = 1), ‘STD-baseline’ (AUC = 0.92) and ‘RMSSD-HR’ (AUC = 0.98) have a high performance, ‘RMSSD-actogram’ (AUC = 0.87) and ‘STD-actogram’ (AUC = 0.86) perform less compared to the other classifier (see Fig. 4b).

### Step 3. Changes over time

#### Development of parameters over gestational age, not divided by fBS

First of all, we tested whether the parameters change with GA regardless the fBS (see supplementary table 3). The parameters did not show any general change during development.

#### Development of parameters over gestational age, divided by fBS

**Figure 5:**
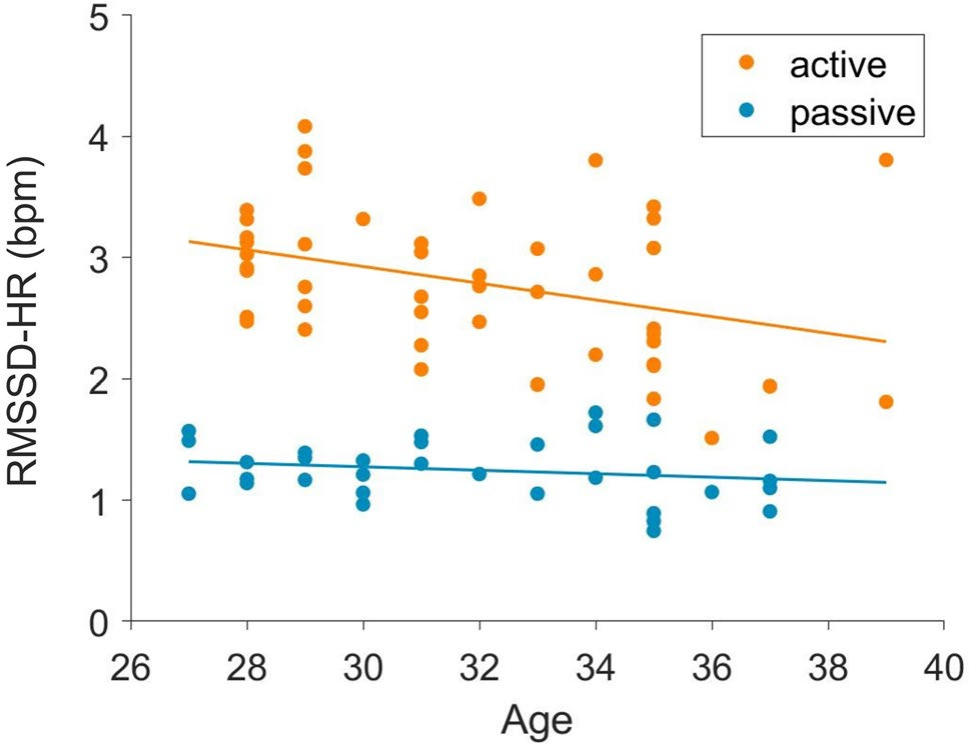
Decrease over gestational age of RMSSD-HR (bpm) in active but not passive fBS.

Furthermore, we investigated whether the parameters change over time depending on the fBS. For this analysis, we combined passive (early passive + 1F) and active (early active + 2F) states to detect their general evolution over time (see supplementary table 4). Only the ‘RMSSD-HR’ showed a difference between states. In particular, the decline can be seen in active states (p = 0.01, r^2^ = 0.13) but not in passive states (p = 0.30; Fig. 5).

#### Development of the correlation cardiogram-actogram over gestational age

**Figure 6:**
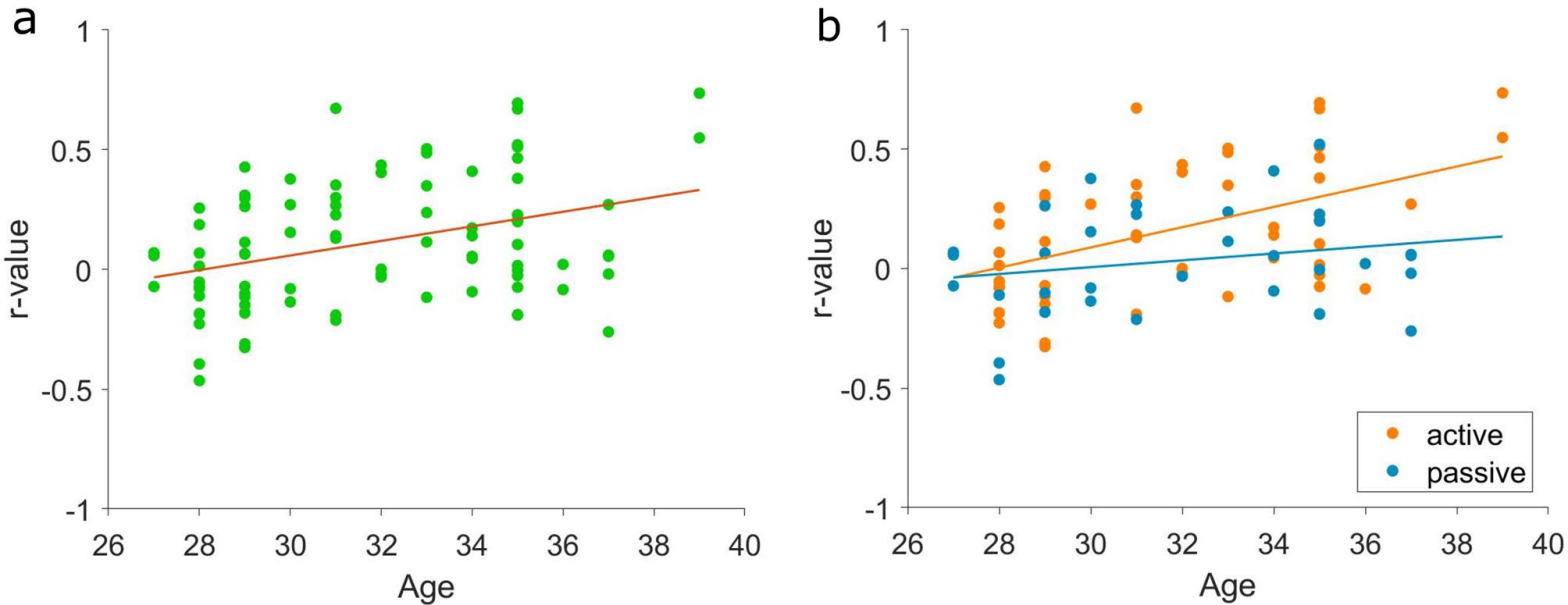
(**a**) Increase of the correlation cardiogram-actogram regardless the fBS. (**b**) This increase is driven by the significant increase of correlation during active states.

Thirdly, we looked at the evolution of the correlation between cardiogram and actogram over time (Fig. 6a). We found an overall increase of the r-values over gestational age (p = 0.001, r^2^ = 0.18).

Introducing the behavioural state as further parameter, we found an increase in the correlation between actogram and cardiogram for active states (p < 0.001, r^2^ = 0.22) but not for passive states (p = 0.2) (Fig. 6b).

## Discussion

This work systematically investigated different parameters commonly used for fBS classification. Although nearly all investigated parameters differed significantly in their distribution between active and passive state, or between 1 and 2F, only a few of these parameters had non-overlapping distributions, preventing a clear separation and classification of the states.

Furthermore, we identified two parameters that continuously changed during fetal development. We therefore argue that they are not suitable for a general fBS classification.

### Step 1. Parameters distribution and thresholds

The first aim of the current work was to investigate the distribution of properties of the acto- and cardiogram over fetal behavioural states. As expected, different parameter distributions are representative for the different states, as confirmed by the significant differences between parameters across states. Moreover, our HRV parameters (mean HR, SDNN-RR and RMSSD-RR) were in line with previous works^9^.

Some emphasis should be put on the parameters ‘% points outside ± 5 bpm’ and ‘% points outside ± 7.5 bpm’. For example, in Brändle et al.^9^, the authors operationalised the variability of the HR considering the oscillation bandwidth in which the HR could vary. Specifically, it is assumed that the HR does not exceed the [baseline ± 7.5] bpm in state 1F. Although this value could efficiently divide between active and passive states during early or late gestation, this threshold was exceeded in each data-window. In states classified as passive, up to 4% of the total data points exceeded this value. This raises the question, whether the threshold value should be increased (e.g. ± 10 bpm), or whether an increase would instead classify active states as passive and vice-versa. Our suggestion would be to define a certain tolerance around this threshold. For example, in our datasets this value could be exceeded in 5% of data points in a passive state and still provide the same results in terms of classification.

Another point is the correlation between the cardiogram and the actogram. In Nijhuis et al.^2^, the authors defined the fBS by means of the different ways heart-rate and body movement relate to each other. As a proxy for this coupling, in our work we explored how heart rate and body movement are related through analysis of the correlation between the two signals. Generally, the correlation was weak, with r-values both positive and negative (Fig. 3). Given the high variability and wide overlap of this parameter over fBS, this parameter does not appear specific of a particular state. However, as discussed later, this index has interesting developmental characteristics.

### Step 2. Best classifier

The ROC analysis revealed that the parameters derived from the HRV have very high classification performance, as noted by the AUC of almost 1 in every case (Fig. 4). Even though movement plays an important role in the initial Nijhuis criteria, the actogram parameters, as defined in the current work, show the worst classification performance with an AUC around 0.80.

Therefore, at least in the frame of fMCG recordings, we argue that the actogram does not improve an automatic fBS classification. However, it should be considered that the actogram only represents gross-body movements and does not represent the movements of extremities.

### Step 3. Changes over time

We found a decrease over gestation in the parameter ‘RMSSD-HR’ in active states but not in passive states (Fig. 5). Even though there is no accordance on the development of short term variability in the heart patterns during the last trimester of gestation, with some works reporting an increase^23^ and others no changes^9^, in our case we found a decrease which depends on the activity state (Fig. 5).

The progressive decline in short-term variability in the heart-rate, as expressed in our findings by the ‘RMSSD-HR’, makes the distinction between active and passive states, based on this parameter, less clear as gestational age increases. This is compatible with evidence for no substantial differences in short term variability in the heart-rate across different sleep stages later in childhood and adolescence^24^.

Furthermore, we found an increasing correlation following gestational age between cardiogram and actogram only in active states (Fig. 6). One reason for the developmental change of this relationship could be the shortening, over gestation, of the lag between fetal heart-rate changes and fetal trunk movement onset. Supporting this hypothesis, for example, Zhao et al.^25^ found that the coupling between these two elements in fact rarely occurs before 36 weeks of GA.

These developmental trends, found in the correlation between cardiogram and actogram, and in the ‘RMSSD-HR’, could reflect the maturational changes of the fetal autonomic nervous system and make these parameters less suitable for fBS analysis over different gestational ages. In particular, these parameters vary not only between early and late gestation, but also within these time frames at least in active states.

### Limitations and outlook

In the present analysis wake states, like 3F and 4F were excluded due to their low occurrence. Including a substantially higher number of datasets could shed light on the parameter distribution over these fBS. Moreover, in contrast to Vairavan et al.^14^, we grouped together fetuses from the middle (32–36 weeks of GA) and late (37–39 weeks of GA) in a whole late group.

Furthermore, the data segments included in the current analysis were selected by a states detection algorithm and validated by two external raters, which results in selecting very prototypical data segments. In the algorithm used in this study (adapted from Vairavan et al.^14^), data segments with a parameter distribution that does not fit the prototypical characteristic of a certain fBS are discarded. Therefore, an algorithm with clearly defined thresholds has a problem with classifying less clear datasets, or datasets with a transition between states. Classification of transitions between wake and sleep states is not only relevant for understanding basic sleep physiology, but also for the study of cognitive processing^26^.

To improve the detection of fBS, one option could be to consider the probability of the fetuses being in one of the fBS compared to the others. In fact, considering the distribution of parameters reported before, it appears that a certain value for a parameter is more likely to occur in a certain state compared to another state. This likelihood could be used for a more dynamic state detection, accounting for transitions. Another approach could then be a dynamic definition of the fBS using the fetal brain data extracted during the measurement. fMEG allows, theoretically, to also use the fetal brain activity as an additional parameter. Even in preterm infants, for example, a state classification based on the electroencephalographic data is at the moment the frontier for automatic state classification^27^.

## Conclusion

Our results indicate that HRV parameters have a higher classification accuracy in comparison to those extracted from the actogram. Therefore, we propose for the automation of fBS detection to concentrate on the HRV instead of body movement as defined by the actogram. Moreover, the changes over time in some of the parameters in our opinion disqualify them from being part of a fBS classification algorithm that simply divides fetuses into early and late groups. In fact, given the parameters continuous change over time, we instead suggest to consider fetal gestational age in weeks. State of the art algorithms, like the one from Vairavan et al.^14^, are easily applicable and partly reflect the original fBS definition criteria. On the other side, these algorithms may be too simplistic, thus failing to grasp more complex physiological events such as transitions between states. Based on these conclusions, our suggestion is therefore the development of a probabilistic approach for the fBS detection that is more dynamic in detecting the alternating patterns between wake and sleep, which could possibly include fetal brain parameters.

## Supplementary Information

Supplementary Information.

## Data Availability

The datasets adopted in the current study are available from the corresponding author on a reasonable request.
