# Supplementary material for: Evaluation of parameters for fetal behavioural state classification
Source: Sci Rep. 2022 Mar 1;12:3410. doi: 10.1038/s41598-022-07476-x (PMC8888564; doi:10.1038/s41598-022-07476-x)
Supplement: Supplementary file 1 — Supplementary Information. [file 41598_2022_7476_MOESM1_ESM.docx]

**Evaluation of parameters for fetal behavioural state classification**

**Supplementary material**

Corresponding author: Lorenzo Semeia

fMEG-Center, Otfried-Müller-Str. 47, 72076 Tübingen (DE)

[lorenzo.semeia@student.uni-tuebingen.de](mailto:lorenzo.semeia@student.uni-tuebingen.de)

Phone: +49 7071 2981192

Co-authors: Dr. Katrin Sippel, Julia Moser, Prof. Dr. Hubert Preissl

fMEG- Center, Otfried-Müller-Str. 47, 72076 Tübingen (DE)

|  | Passive | Active |  |
| --- | --- | --- | --- |
| HRV parameters |  |  | **p value** |
| Mean HR | 138.04 ± 3.96 | 140.60 ± 6.94 | P = 0.15 |
| SDNN | 9.86 ± 2.21 | 20.72 ± 4.68 | p < 0.001 |
| RMSSD | 4.37 ± 0.87 | 9.65 ± 2.09 | p < 0.001 |
| Cardiogram parameters |  |  |  |
| Baseline std | 1.95 ± 0.67 | 3.68 ± 1.20 | p < 0.001 |
| % points outside ± 5 bpm | 6.36 ± 2.21 | 32.76 ± 7.88 | p < 0.001 |
| % points outside ± 7.5 bpm (%) | 1.57 ± 1.07 | 18.25 ± 4.77 | p < 0.001 |
| RMSSD in the HR | 1.28 ± 0.18 | 2.96 ± 0.49 | p < 0.001 |
| HR std | 3.51 ± 0.81 | 7.72 ± 1.12 | p < 0.001 |
| Actogram parameters |  |  |  |
| RMSSD in the actogram | 0.07 ± 0.03 | 0.11 ± 0.04 | p = 0.001 |
| Actogram std | 0.16 ± 0.06 | 0.26 ± 0.08 | p < 0.001 |

***Supplementary Table 1.*** *Parameters values* *[mean ± standard deviation] for early gestation (less than 32 weeks of GA).*

|  | 1F | 2F |  |
| --- | --- | --- | --- |
| HRV parameters |  |  | **p value** |
| Mean HR | 137.47 ± 10.88 | 138.63 ± 7.51 | p = 0.70 |
| SDNN | 8.32 ± 2.77 | 20.10 ± 5.74 | p < 0.001 |
| RMSSD | 3.64 ± 1.05 | 9.10 ± 3.17 | p < 0.001 |
| Cardiogram parameters |  |  |  |
| Baseline std | 1.47 ± 0.58 | 3.77 ± 1.55 | p < 0.001 |
| % points outside ± 5 bpm | 4.75 ± 4.41 | 31.51 ± 4.80 | p < 0.001 |
| % points outside ± 7.5 bpm | 1.34 ± 1.49 | 18.56 ± 3.99 | p < 0.001 |
| RMSSD in the HR | 1.21 ± 0.32 | 2.56 ± 0.69 | p < 0.001 |
| HR std | 3.00 ± 1.05 | 7.78 ± 1.29 | p < 0.001 |
| Actogram parameters |  |  |  |
| RMSSD in the actogram | 0.04 ± 0.03 | 0.11 ± 0.06 | p < 0.001 |
| Actogram std | 0.14 ± 0.09 | 0.36 ± 0.19 | p < 0.001 |

***Supplementary table 2.*** *Parameters values* *[mean ± standard deviation] for late gestation (from 33 weeks of GA).*

|  | p-value |
| --- | --- |
| Cardiogram parameters |  |
| Baseline std | p = 0.63 |
| % points outside ± 5 bpm | p = 0.26 |
| % points outside ± 7.5 bpm | p = 0.76 |
| RMSSD in the HR | p = 0.12 |
| HR std | p = 0.67 |
| Actogram parameters |  |
| RMSSD in the actogram | p = 0.23 |
| Actogram std | p = 0.40 |

***Supplementary table 3.*** *p-values corresponding to the linear models underlying each of the parameter regardless the fBS. The models test whether the different parameters change with gestational age.*

|  | Passive | Active |
| --- | --- | --- |
| Cardiogram parameters |  |  |
| Baseline std | p = 0.07 | p = 0.93 |
| % points outside ± 5 bpm | p = 0.94 | p = 0.52 |
| % points outside ± 7.5 bpm | p = 0.37 | p = 0.70 |
| RMSSD in the HR | p = 0.30 | p = 0.01 |
| HR std | p = 0.06 | p = 0.67 |
| Actogram parameters |  |  |
| Actogram std | p = 0.31 | p = 0.09 |
| RMSSD in the actogram | p = 0.06 | p = 0.67 |

***Supplementary table 4.*** *p-values corresponding to the linear models underlying each of the parameter divided by fBS.*
